# Annual acknowledgement of manuscript reviewers

**DOI:** 10.1186/s13068-015-0198-y

**Published:** 2015-03-28

**Authors:** Michael E Himmel, James du Preez, Debra Mohnen, Charles Wyman

**Affiliations:** Chemical Biosciences Center, National Renewable Energy Laboratory, 15013, Denver, West Parkway, Golden, 80401 CO USA; Department of Microbial, Biochemical and Food Biotechnology, University of the Free State, PO Box 339, Bloemfontein, 9300 South Africa; Department of Biochemistry and Molecular Biology, Complex Carbohydrate Research Center, University of Georgia, Athens, 30602 GA USA; Department of Chemical and Environmental Engineering, Bourns College of Engineering, University of California, 900 University Avenue, Riverside, 92521 CA USA

## Abstract

The editors of *Biotechnology for Biofuels* would like to thank all our reviewers who have contributed to the journal in Volume 7 (2014).

**Gabriel Acien**

Spain

**Dilip Adhikari**

India

**William Adney**

United States of America

**George Aggelis**

Greece

**M. Kalim Akhtar**

United Kingdom

**Eva Albers**

Sweden

**João R.M. Almeida**

Brazil

**Hal Alper**

United States of America

**Jean Alric**

France

**Diletta Ami**

Italy

**Danuta Maria Antosiewicz**

Poland

**Ana Arabolaza**

Argentina

**Patricia Armshaw**

Ireland

**Frances Arnold**

United States of America

**Vincent Arondel**

France

**Mikko Arvas**

Finland

**Shota Atsumi**

United States of America

**Claudio Avignone-Rossa**

United Kingdom

**Hyeun-Jong Bae**

Korea, South

**Fengwu Bai**

China

**John Baker**

United States of America

**Scott Baker**

United States of America

**Robert Bakker**

Netherlands

**Venkatesh Balan**

United States of America

**Petr Baldrian**

Czech Republic

**Mercedes Ballesteros**

Spain

**Narcisa Bandarra**

Portugal

**Jie Bao**

China

**Brian Barr**

United States of America

**Amanda Barry**

United States of America

**Roberto Bassi**

Italy

**Jacqueline Batley**

Australia

**Khorcheska Batyrova**

Canada

**Stefan Bauer**

United States of America

**Gregg Beckham**

United States of America

**Isabel Belo**

Portugal

**John Benemann**

United States of America

**J. Philipp Benz**

Germany

**Bryan Berger**

United States of America

**Nicolas Bernet**

France

**Jean-Guy Berrin**

France

**Alexandre Besson**

France

**Aaron Best**

United States of America

**Maurizio Bettiga**

Sweden

**Samarthya Bhagia**

United States of America

**Balasubramaniyan Bharathiraja**

India

**Peter Biely**

Slovakia

**Natascia Biondi**

Italy

**Kenneth Bischoff**

United States of America

**Ajaya Biswal**

United States of America

**Hans Blaschek**

United States of America

**Wout Boerjan**

Belgium

**Rafal Bogel-Lukasik**

Portugal

**Sivia Bolado**

Spain

**Eckhard Boles**

Germany

**Yannick Bomble**

United States of America

**Elba Bon**

Brazil

**Nicklas Bonander**

Sweden

**Antonio Bonomi**

Brazil

**Mallika Boonmee**

Thailand

**Ilya Borovok**

Israel

**Suncica Bosak**

Croatia

**Federica Brandizzi**

United States of America

**Paola Branduardi**

Italy

**Nicholas Brereton**

United Kingdom

**Orianna Bretschger**

United States of America

**Guido Breuer**

Netherlands

**Jeff Broadbent**

United States of America

**Steven Brown**

United States of America

**Harry Brumer**

Canada

**Phillip Brumm**

United States of America

**Tim Bugg**

United Kingdom

**Vincent Bulone**

Sweden

**Renata Bura**

United States of America

**Zeynep Petek Cakar**

Turkey

**Thomas Canam**

United States of America

**Danilo A. Cantero**

Spain

**Carlos Ariel Cardona**

Colombia

**Helene Carrere**

France

**Julie Carrier**

United States of America

**Serge Casaregola**

France

**Eulogio Castro**

Spain

**María del Carmen Cerón-García**

Spain

**Anuj Chandel**

Brazil

**Jo-Shu Chang**

Taiwan

**Jongsoo Chang**

Korea, South

**Afroditi Chatzifragkou**

United Kingdom

**Shuqing Chen**

China

**Hongzhang Chen**

China

**Ning Chen**

China

**Rachel Chen**

United States of America

**Xiaowen Chen**

United States of America

**Kit Ling Chin**

Malaysia

**Paul Christakopoulos**

Sweden

**Panagiotis Chrysanthopoulos**

Australia

**Marek Cieplak**

Poland

**Alan Collmer**

United States of America

**Lydia Contreras**

United States of America

**Qiu Cui**

China

**Jean-Marc Daran**

Netherlands

**Al Darzins**

United States of America

**Gideon Davies**

United Kingdom

**Marcos Antonio de Morais Jr**

Brazil

**Pascale de Philip**

France

**Ronald de Vries**

Netherlands

**Steve Decker**

United States of America

**Luis Del Rio**

Canada

**Matthew DeLisa**

United States of America

**Marie Demuez**

Spain

**Santino Di Berardino**

Portugal

**Bruce Dien**

United States of America

**Shi-You Ding**

United States of America

**Elio Dinuccio**

Italy

**Davide Dionisi**

United Kingdom

**Richard Dixon**

United States of America

**Lucilia Domingues**

Portugal

**Bryon Donohoe**

United States of America

**Jennifer Dunn**

United States of America

**Robert Dunn**

United States of America

**Vincent Eijsink**

Norway

**Thomas Elder**

United States of America

**James Elkins**

United States of America

**Stacia Engel**

United States of America

**Marta Esteban Clares**

Spain

**Thaddeus Ezeji**

United States of America

**Jinxia Fan**

China

**Ming Fan**

Canada

**Xu Fang**

China

**JianMin fang**

United Kingdom

**Pedram Fatehi**

Canada

**Claus Felby**

Denmark

**Bruno Fernandes**

Portugal

**Francisco Jesus Fernandez Morales**

Spain

**Roberto Fernandez-Lafuente**

Spain

**Manuel Ferrer**

Spain

**Oliver Fiehn**

United States of America

**Oliver Fiehn**

Germany

**Henri-Pierre Fierobe**

France

**Stephen Fong**

United States of America

**César Fonseca**

Portugal

**Luis Fonseca**

Portugal

**Marcus Foston**

United States of America

**Eric Fouilland**

France

**Brian Fox**

United States of America

**Jean Marie Francois**

France

**Annaliese Franz**

United States of America

**Ruth Freitag**

Germany

**Todd French**

United States of America

**Stephen C. Fry**

United Kingdom

**Tatsuhito Fujimura**

Japan

**Eiichiro Fukusaki**

Japan

**Mats Galbe**

Sweden

**Anli Geng**

Singapore

**Fernando Genta**

Brazil

**Richard Giannone**

United States of America

**Thierry Giardina**

France

**Donna Gibson**

United States of America

**Notburga Gierlinger**

Austria

**Harry Gilbert**

United Kingdom

**Francisco Girio**

Portugal

**Rosana Goldbeck**

Brazil

**Leonardo Gomez**

United Kingdom

**Raquel B. Gómez-Coca**

Spain

**Katsuya Gomi**

Japan

**Paula Goncalves**

Portugal

**Cristina Gonzalez-Fernandez**

Spain

**Ursula Goodenough**

United States of America

**Johann Gorgens**

South Africa

**Steven Gorsich**

United States of America

**Hugo Gramajo**

Argentina

**Melinda Griffiths**

South Africa

**Bernhard Grimm**

Germany

**Dominique Grizeau**

France

**Amy Grunden**

United States of America

**Michael Guarnieri**

United States of America

**Luis Tiago Guerra**

Portugal

**Fabienne Guillon**

France

**Stephane Guillouet**

France

**Gia-Luen Guo**

Taiwan

**Vijay Kumar Gupta**

Ireland

**Ana Gutierrez**

Spain

**Yitzhak Hadar**

Israel

**Patrick Hallenbeck**

Canada

**Jason Hallett**

United Kingdom

**Claire Halpin**

United Kingdom

**Sung Ok Han**

Korea, South

**Paul Harris**

United States of America

**Annele Hatakka**

Finland

**Mingxiong He**

China

**Lucy Heap**

United Kingdom

**Ronald Hector**

United States of America

**Eric Hegg**

United States of America

**Juergen Heinisch**

Germany

**Klaas Jan Hellingwerf**

Netherlands

**Bernard Henrissat**

France

**Michael Henson**

United States of America

**Isabelle Herpoël-Gimbert**

France

**Robert Hettich**

United States of America

**Mark Hildebrand**

United States of America

**Serge Hiligsmann**

Belgium

**Maria Hingsamer**

Austria

**David Hodge**

United States of America

**Seong Joo Hong**

Korea, South

**Svein Jarle Horn**

Norway

**Lihua Hou**

China

**Bo Hu**

United States of America

**Jinguang Hu**

Canada

**Hong-Ying Hu**

China

**Paul Hudson**

Sweden

**Patrick Hunt**

United States of America

**David Ibarra**

Spain

**Lonnie Ingram**

United States of America

**Hiroyuki Inoue**

Japan

**Makio Iwahashi**

Japan

**Javier Izquierdo**

United States of America

**Sebastian Jaenicke**

Germany

**Neeraj Jain**

India

**Sarath Chandra Janga**

United States of America

**Christer Jansson**

United States of America

**Kaemwich Jantama**

Thailand

**Grzegorz Janusz**

Poland

**Thomas Jeffries**

United States of America

**Dieter Jendrossek**

Germany

**Tina Jeoh**

United States of America

**Mingjie Jin**

United States of America

**Yong-Su Jin**

United States of America

**Vladimir Jiranek**

Australia

**Katja Salomon Johansen**

Denmark

**Sussai John Vennison**

India

**Wayne Johnston**

Australia

**Patrik Jones**

United Kingdom

**Henning Jorgensen**

Denmark

**Yi-Hsu Ju**

Taiwan

**Uldis Kalnenieks**

Latvia

**Yoichi Kamagata**

Japan

**Katy Kao**

United States of America

**Levente Karaffa**

Hungary

**Dimitar Karakashev**

Denmark

**Kaisa Karhumaa**

Sweden

**Baljinder Kaur**

India

**Katariina Kemppainen**

Finland

**Christian Kennes**

Spain

**Sung-Koo Kim**

Korea, South

**Seon-Won Kim**

Korea, South

**Sylvia Klaubauf**

Sweden

**Mario Klimacek**

Austria

**Keith Kline**

United States of America

**Paul Knox**

United Kingdom

**Caner Koc**

Turkey

**Konrad Koch**

Germany

**Kari Koivuranta**

Finland

**Akihiko Kondo**

Japan

**Yoon-Mo Koo**

Korea, South

**Maarten Kootstra**

Netherlands

**Rakesh Koppram**

Austria

**Katalin Kovacs**

United Kingdom

**Krisztina Kovacs**

Sweden

**Kornél L. Kovács**

Hungary

**Andrey Kovalevsky**

United States of America

**Preben Krabben**

United Kingdom

**Emma Kreuger**

Sweden

**Michael Krogh Jensen**

Denmark

**Peter Kroth**

Germany

**Nathan Kruer-Zerhusen**

United States of America

**Olaf Kruse**

Germany

**Malgorzata Krzywonos**

Poland

**Christian P. Kubicek**

Austria

**Ramesh Chander Kuhad**

India

**Ravindra Kumar**

India

**Linoj Kumar**

Canada

**Rajeev Kumar**

United States of America

**Suresh Kumar**

India

**Taís Mayumi Kuniyoshi**

Brazil

**Michael Ladisch**

United States of America

**Aino-Maija Lakaniemi**

Finland

**Jerald Lalman**

Canada

**Alicia Lammerts van Bueren**

Netherlands

**Louis Landesman**

United States of America

**Nuno Lapa**

Portugal

**Magin Lapuerta**

Spain

**Edmundo Larenas**

United States of America

**Johan Larsbrink**

Norway

**Christer Larsson**

Sweden

**Lieve Laurens**

United States of America

**Dong-Yup Lee**

Singapore

**Keat Teong Lee**

Malaysia

**Eun Yeol Lee**

Korea, South

**Hung Lee**

Canada

**Sang Yup Lee**

Korea, South

**Sang Hun Lee**

United States of America

**Seokhwan Lee**

Korea, South

**Taek Soon Lee**

United States of America

**Y. Y. Lee**

United States of America

**Gustavo Leite**

Canada

**Guillaume Letellier**

France

**Frederick Leung**

Hong Kong

**David Levin**

Canada

**Orly Levitan**

United States of America

**Chenlin Li**

United States of America

**Jian-Jun Li**

China

**Shengying Li**

China

**Pengfu Li**

China

**Robert Li**

United States of America

**Si-Yu Li**

Taiwan

**Shizhong Li**

China

**Yan Li**

Australia

**Yantao Li**

United States of America

**Yuanguang Li**

China

**Yonghua Li-Beisson**

France

**Gunnar Liden**

Sweden

**Carol Sze Ki Lin**

Hong Kong

**Senjie Lin**

United States of America

**Philipp Lins**

Austria

**Dehua liu**

China

**Yan Liu**

United States of America

**Z. Lewis Liu**

United States of America

**Irakli Loladze**

United States of America

**Teresa Lopes da Silva**

Portugal

**Isabel M. Lopez-Lara**

Mexico

**John Love**

United Kingdom

**Cândida Lucas**

Portugal

**Roland Ludwig**

Austria

**Andriy Luzhetskyy**

Germany

**Lee Lynd**

United States of America

**Kesen Ma**

Canada

**Xiaojun Ma**

China

**Warren Mabee**

Canada

**Robert L. Mach**

Austria

**Robert Mach**

Austria

**Alasdair Mackenzie**

Norway

**Rod Mackie**

United States of America

**Stefano Macrelli**

Sweden

**Nirupama Mallick**

India

**Silvio Mangini**

Italy

**Paloma Manzanares**

Spain

**Christos Maravelias**

United States of America

**Antoine Margeot**

France

**Isabel Marques**

Portugal

**Paula Marques**

Portugal

**Mark Marten**

United States of America

**Vincent Martin**

Canada

**Alfredo Martinez**

Mexico

**Alain Marty**

France

**Emma Master**

Canada

**Roberto Mazzoli**

Italy

**Richard Meilan**

United States of America

**Rodrigo Mendes**

Brazil

**Angel Merida**

Spain

**Anne S. Meyer**

Denmark

**Vera Meyer**

Germany

**Yun-gen Miao**

China

**Rowan A.C. Mitchell**

United Kingdom

**Eric Moellering**

United States of America

**Debra Mohnen**

United States of America

**Emilio Molina Grima**

Spain

**Frederic Monot**

France

**Beronda Montgomery**

United States of America

**Stephen Moose**

United States of America

**Ely Morag**

Israel

**Jose Moran-Mirabal**

Canada

**José Luis Moreno**

Spain

**Haruhide Mori**

Japan

**Melanie Mormile**

United States of America

**Uffe Mortensen**

Denmark

**Nathan Mosier**

United States of America

**Patrícia Moura**

Portugal

**Sujatha Mulpuri**

India

**Andrew Munro**

United Kingdom

**Koenraad Muylaert**

Belgium

**Nick Nagle**

United States of America

**Endre Nagy**

Hungary

**Satish Nair**

United States of America

**Kazunori Nakashima**

Japan

**Shunichi Nakayama**

Japan

**Jarunya Narangajavana**

Thailand

**Neelakantam Narendranath**

United States of America

**Donald Natvig**

United States of America

**Jose Nery**

Brazil

**William Nes**

United States of America

**Helena Nevalainen**

Australia

**Elke Nevoigt**

Germany

**Jean-Marc Nicaud**

France

**Nancy Nichols**

United States of America

**Bernd Nidetzky**

Austria

**Catherine Niu**

Canada

**Beatriz Nobre**

Portugal

**Sue Nokes**

United States of America

**Intawat Nookaew**

Sweden

**Joakim Norbeck**

Sweden

**Birgir Norddahl**

Denmark

**Toshihiro Obata**

Germany

**Michael O'Donohue**

France

**Kyeong Keun Oh**

Korea, South

**Min-Kyu Oh**

Korea, South

**Piotr Oleskowicz-Popiel**

Poland

**Jose Miguel Oliva**

Spain

**Ana Cristina Oliveira**

Portugal

**Marina Oliveira de Souza Dias**

Brazil

**Kim Olofsson**

Denmark

**Daniel Olson**

United States of America

**Benny Palmqvist**

Sweden

**Xuejun Pan**

United States of America

**Seraphim Papanikolaou**

Greece

**E. Terry Papoutsakis**

United States of America

**Prathap Parameswaran**

United States of America

**Volkmar Passoth**

Sweden

**Francisco I. Javier Pastor**

Spain

**Gilles Peltier**

France

**John Peters**

United States of America

**Loukas Petridis**

United States of America

**Dimitris Petroutsos**

France

**Riikka Piispanen**

Finland

**Domenico Pirozzi**

Italy

**Jon Pittman**

United Kingdom

**Jandora Poli**

Brazil

**Phillip Pope**

Norway

**Matthew Posewitz**

United States of America

**Antje Potthast**

Austria

**Robert Powers**

United States of America

**Rolf Prade**

United States of America

**Kristala Prather**

United States of America

**Jeremy Pruvost**

France

**Yuqi Qin**

China

**Yinbo Qu**

China

**Sathishkumar Ramalingam**

India

**Arthur Ragauskas**

United States of America

**Jenni Rahikainen**

Finland

**Arthur F.J. Ramachandriya**

Netherlands

**Karthikeyan Dharman**

United States of America

**Luiz Ramos**

Brazil

**Roberto Rana**

Italy

**Reza Ranjbar**

Netherlands

**Aaron Rashotte**

United States of America

**Eric Record**

France

**Ghufran Redzwan**

Malaysia

**Jennifer Reed**

United States of America

**Lars Rehmann**

Canada

**Peter Richard**

Finland

**Hanno Richter**

United States of America

**Daniel Rigden**

United Kingdom

**Barbara Rincon**

Spain

**Jukka Rintala**

Finland

**Isabel Rocha**

Portugal

**Paul Roessler**

United States of America

**Peter Rogers**

Australia

**Manuela Rollini**

Italy

**Jose Romero**

Spain

**Shaunita Rose**

South Africa

**Alexander Ruban**

United Kingdom

**Wattiez Ruddy**

Belgium

**Anne Ruffing**

United States of America

**Anas Saadeddin**

Spain

**Jack Saddler**

Canada

**Daad Saffarini**

United States of America

**Kazuo Sakka**

Japan

**Deanne Sammond**

United States of America

**Mats Sandgren**

Sweden

**Anders Sandström**

Sweden

**Sanjaya**

United States of America

**Frank Sargent**

United Kingdom

**Ilona Sarvari Horvath**

Sweden

**Hisashi Satoh**

Japan

**Luc Saulnier**

France

**Shigeki Sawayama**

Japan

**Gary Sawers**

Germany

**Christopher Scarlata**

United States of America

**Michael Scharf**

United States of America

**Henrik Scheller**

United States of America

**Bettina Schiel-Bengelsdorf**

Germany

**Jonathan Schilling**

United States of America

**Andreas Schirmer**

United States of America

**Robert Schmidt**

United States of America

**Monika Schmoll**

Austria

**Anna Schnürer**

Sweden

**Johannes Scholten**

United States of America

**Augusto Schrank**

Brazil

**Kathrin Schrick**

United States of America

**Wolfgang Schwarz**

Germany

**Alexander Sczyrba**

Germany

**Bernhard Seiboth**

Austria

**Michael Selig**

Denmark

**Luis Serrano**

Spain

**Osman Ugur Sezerman**

Turkey

**A. Joe Shaw**

United States of America

**Reshma Shetty**

United States of America

**Haifeng Shi**

United States of America

**Jian Shi**

United States of America

**Hojae Shim**

Macao

**Hyun-Dong Shin**

United States of America

**Verena Siewers**

Sweden

**Jean-Claude Sigoillot**

France

**Pedro Simões**

Portugal

**Steven Singer**

United States of America

**Munir Skaf**

Brazil

**Kelly Skinner**

United States of America

**Raphael Slade**

United Kingdom

**John Slattery**

United Kingdom

**Emily Smith**

United States of America

**Hyohak Song**

Korea, South

**Morten Sørlie**

Norway

**Ana Maria Souto-Maior**

Brazil

**Riccardo Spaccini**

Italy

**Richard Sparling**

Canada

**Alfons Stams**

Netherlands

**Eric Steen**

United States of America

**Jonathan Stickel**

United States of America

**Gerlind Sulzenbacher**

France

**Run-Cang Sun**

China

**Sandhya Swaminathan**

India

**Edyta Szewczyk**

United States of America

**Eriko Takano**

United Kingdom

**Yue-Qin Tang**

China

**Yinjie J. Tang**

United States of America

**Sarah Teter**

United States of America

**Johan Thevelein**

Belgium

**Leya Thomas**

India

**Chaoguang Tian**

China

**Ming Tien**

United States of America

**Folke Tjerneld**

Sweden

**Elia Tomas-Pejo**

Spain

**Giuseppe Torzillo**

Italy

**Tina Tran**

Australia

**Karen Trchounian**

Armenia

**Armen Trchounian**

Armenia

**Cong Trinh**

United States of America

**Maobing Tu**

United States of America

**Gregory Tucker**

United Kingdom

**Wallace Tyner**

United States of America

**Cirano Ulhoa**

Brazil

**Daisuke Umeno**

Japan

**Breeanna Urbanowicz**

United States of America

**Jean-Louis Uribelarrea**

France

**Mari Valkonen**

Finland

**Johan Van Groenestijn**

Netherlands

**Antonius van Maris**

Netherlands

**Jolanda van Munster**

United Kingdom

**Ed van Niel**

Sweden

**Dries Vandamme**

Belgium

**Herve Vanderschuren**

Switzerland

**Marco Vanoni**

Italy

**Cristian Varela**

Australia

**Joao Varela**

Portugal

**Arul Mozhy Varman**

United States of America

**Anikó Várnai**

Norway

**Dimitris Vayenas**

Greece

**Rafael Vazquez-Duhalt**

Mexico

**Wim Vermaas**

United States of America

**Simone Vincenzi**

Italy

**Jaap Visser**

Netherlands

**Heinrich Volschenk**

South Africa

**Keith Waldron**

United Kingdom

**Larry Walker**

United States of America

**Ola Wallberg**

Sweden

**Dirk Walther**

Germany

**Chengshu Wang**

China

**Michael Wang**

United States of America

**Wei Wang**

China

**Zhongming Wang**

China

**Weiliang Wang**

China

**Xin Wang**

China

**Hui Wei**

United States of America

**Paul Weimer**

United States of America

**Bjorge Westereng**

Norway

**Peter Westh**

Denmark

**Janet Westpheling**

United States of America

**Christian Wilhelm**

Germany

**David Wilson**

United States of America

**Gideon Wolfaardt**

Canada

**Aaron Wright**

United States of America

**Mark Wright**

United States of America

**Jin Chuan Wu**

Singapore

**Guojiang Wu**

China

**Mo Xian**

China

**Eduardo Ximenes**

United States of America

**Xin-Hui Xing**

China

**Bingqian Xu**

United States of America

**Jian Xu**

China

**Zeng-Fu Xu**

China

**Shihui Yang**

United States of America

**Sheng Yang**

China

**Hong-Wei Yen**

Taiwan

**Carl Yeoman**

United States of America

**Minyeong Yoo**

France

**Ho-Sung Yoon**

Korea, South

**Chun You**

United States of America

**Jiujiang Yu**

United States of America

**Joshua Yuan**

United States of America

**Qipeng Yuan**

China

**Guido Zacchi**

Sweden

**An-Ping Zeng**

Germany

**Min Zhang**

United States of America

**Xiao Zhang**

United States of America

**Y-H Percival Zhang**

United States of America

**Ximing Zhang**

China

**Guang Zhao**

China

**Zongbao Zhao**

China

**Jizhong Zhou**

United States of America

**Zhi-Gang Zhou**

China

**Zhihua Zhou**

China

**Francesco Zimbardi**

Italy

**Min-Hua Zong**

China

